# Gene sharing among plasmids and chromosomes reveals barriers for antibiotic resistance gene transfer

**DOI:** 10.1098/rstb.2020.0467

**Published:** 2022-01-17

**Authors:** Yiqing Wang, Aditi Batra, Hinrich Schulenburg, Tal Dagan

**Affiliations:** ^1^ Institute of General Microbiology, Kiel University, Kiel, Germany; ^2^ Zoological institute, Kiel University, Kiel, Germany

**Keywords:** horizontal gene transfer, antibiotic resistance, *Escherichia*, *Klebsiella*, *Salmonella*

## Abstract

The emergence of antibiotic resistant bacteria is a major threat to modern medicine. Rapid adaptation to antibiotics is often mediated by the acquisition of plasmids carrying antibiotic resistance (ABR) genes. Nonetheless, the determinants of plasmid-mediated ABR gene transfer remain debated. Here, we show that the propensity of ABR gene transfer via plasmids is higher for accessory chromosomal ABR genes in comparison with core chromosomal ABR genes, regardless of the resistance mechanism. Analysing the pattern of ABR gene occurrence in the genomes of 2635 Enterobacteriaceae isolates, we find that 33% of the 416 ABR genes are shared between chromosomes and plasmids. Phylogenetic reconstruction of ABR genes occurring on both plasmids and chromosomes supports their evolution by lateral gene transfer. Furthermore, accessory ABR genes (encoded in less than 10% of the chromosomes) occur more abundantly in plasmids in comparison with core ABR genes (encoded in greater than or equal to 90% of the chromosomes). The pattern of ABR gene occurrence in plasmids and chromosomes is similar to that in the total *Escherichia* genome. Our results thus indicate that the previously recognized barriers for gene acquisition by lateral gene transfer apply also to ABR genes. We propose that the functional complexity of the underlying ABR mechanism is an important determinant of ABR gene transferability.

This article is part of the theme issue ‘The secret lives of microbial mobile genetic elements’.

## 1. Introduction

Bacteria harbour vast potential to rapidly adapt to different selective environments, including environments defined by antibiotics. Notably, antibiotic resistance (ABR) predates the clinical use of antibiotics: ABR genes have been reported from permafrost [1] and ancient human remains [2]. Today, the ability of bacteria to adapt to antibiotics forms the core of the ABR crisis [3]. Drug therapy is aimed at killing bacteria by targeting key components and processes that are crucial for their reproduction; the repertoire of genetic variants giving rise to ABR therefore includes many core genes [4]. For example, bacterial efflux pumps, which function in resistance to diverse antibiotics, have multiple roles in bacterial physiology in addition to removal of antibiotics from the cell, including the extrusion of heavy metals and cell signalling molecules [5]. Indeed, genes encoding efflux pumps and their regulation are typically part of bacterial core genomes, i.e. they are nearly universally present in bacterial genomes [6]. By contrast, other resistance mechanisms are encoded by genes in the accessory genome and can be found also on mobile genetic elements (e.g. genes involved in target replacement or target protection) [7]. While the emergence of ABR under long-term drug therapy is common via de novo mutations, lateral transfer of ABR genes by mobile genetic elements has the potential to disseminate resistance to various antibiotics within microbial communities in clinical settings, agriculture and also the environment [8,9].

The acquisition of ABR genes can be mediated by three main transfer mechanisms: transformation, transduction and conjugation. Bacterial organisms that are naturally competent may acquire ABR genes via natural transformation of mobile genetic elements carrying ABR genes, including transposons, insertion sequence (IS) elements and integrons (e.g. [10]). Transduction can cause the transfer of ABR genes encoded in the genome of lysogenic phages (e.g. [11,12]) or the transfer of ABR gene-containing plasmids by phages (e.g. [13]). Notwithstanding, plasmid-mediated ABR gene transfer occurs most commonly via conjugation, which facilitates the transfer of conjugative and mobilizable plasmids (e.g. [14]). The dissemination of ABR genes by plasmids is often associated with the evolution of plasmid-encoded integrons that may comprise multiple ABR genes [15]. The type of vehicle that meditates the ABR gene transfer may have implications for the phylogenetic range of the transfer event. Phage-mediated gene transfer is often restricted to closely related donors and recipients owing to phage host specificity [12]. Similarly, the apparent taxonomic pattern in networks of plasmid sequence similarity [16] suggests that plasmid host range constrains the range of plasmid-mediated gene transfer to closely related donor and recipients (with the exception of broad host range plasmids, e.g. [17]).

The prevalence of gene transfer may furthermore depend on genetic and functional characteristics of the transferred gene. Genes that encode proteins whose function depends on interaction with multiple proteins, e.g. within the same complex or cellular process, are rarely transferred, likely because their acquisition leads to interference with the component sociometry [18,19]. An increase in gene copy number following lateral gene transfer may often lead to disadvantageous dose effect, which has been shown to pose a barrier to lateral gene transfer [20,21]. The acquisition of novel laterally transferred genes may have deleterious effects on the recipient owing to diverse reasons ranging from the consequences of foreign DNA integration into the genome to proper expression and function of the protein product in the cell (e.g. [22]; reviewed in [23]). A potential disruption of chromosomal architecture may be minimized if genes are transferred by and expressed from extra-chromosomal genetic elements like plasmids (i.e. assuming non-integrative plasmids). Nonetheless, ABR gene expression from a plasmid locus may be accompanied by fitness cost to the host owing to various other reasons. For example, ABR mechanisms that rely on drug inactivation may require a lower energetic investment (i.e. in ATP) of the cell in comparison with efflux pumps [24]. Such trade-offs in the adaptation of ABR plasmids in newly colonized hosts are expected to have consequences for the evolution of plasmid ABR gene content.

Plasmid gene content is furthermore expected to be subject to purifying selection owing to genetic conflicts between plasmid and chromosomally encoded loci that affect the same phenotype [25]. Indeed, a recent study suggests that the evolution of plasmid ABR gene content depends on the level of genetic conflicts with chromosomally encoded genes, which is strongest for ABR genes encoding efflux pumps and weakest for ABR genes encoding antibiotic target inactivation mechanisms [7]. Considering the association between ABR gene classification into core or accessory genome and the ABR mechanism, we hypothesize that the prevalence of ABR genes on plasmids depends on the ABR gene occurrence in bacterial chromosomes. Here, we examine ABR gene occurrence in bacterial plasmids and chromosomes using a large-scale analysis of three Enterobacteriaceae genera: *Escherichia*, *Salmonella* and *Klebsiella*, for which a large number of genomes are publicly available. We further assess whether ABR gene occurrence on plasmids and chromosomes is associated with the resistance mechanism and inferred ABR gene transfer events using phylogenetics and the temporal pattern of gene occurrence in plasmids and chromosomes.

## 2. Data and methods

To study the pattern of ABR gene occurrence in bacterial replicons (chromosomes and plasmids), we examined all complete genomes of plasmid-carrying isolates in the genera *Escherichia*, *Salmonella* and *Klebsiella* from the NCBI genome database (electronic supplementary material, table S1). Assembly and annotation files of 2580 genomes were downloaded from the NCBI RefSeq database [26] (v. 01/2021). An additional 78 complete genomes lacking RefSeq annotation were downloaded from the GenBank database. Protein-coding genes of the 78 genomes were annotated using Prokka [27] (v. 1.14.6, with parameter --kingdom Bacteria --gcode 11 --genus (Escherichia/Salmonella/Klebsiella) --usegenus --evalue 1 × 10^−9^). Isolate metadata were downloaded from the BioSample database. Samples lacking host information were examined in detail. A total of 23 strains sequenced within the framework of laboratory experiments were excluded. The surveyed genomes include: 1169 chromosomes and 3286 plasmids from *Escherichia*; 846 chromosomes and 3020 plasmids from *Klebsiella*; 620 chromosomes and 1090 plasmids from *Salmonella*. Homologues to genes that may provide ABR were identified based on the Comprehensive Antibiotic Resistance Database (CARD; v. 3.1.0) using the Resistance Gene Identifier tool (RGI; v. 5.1.1, with parameter --clean) [28]. The application of RGI uncovered 126 910 protein-coding genes; of these, we excluded 4774 protein-coding genes whose sequence had less than 70% identity or was less than 90% shorter or greater than 30% longer in comparison with the CARD ABR genes. Of the total 3044 genes in CARD, 416 (14%) ABR genes had 122 136 homologues in the examined isolates. Of those, we further excluded 9 genes belonging to multiple ABR mechanism classes.

The inference of protein families in *Escherichia* was performed using a dataset of 599 completely sequenced *Escherichia* strains downloaded from the NCBI RefSeq database (v. 2018) (previously reported in [29]). Of the total strains, 416 plasmid-carrying isolates were retained for further analysis (electronic supplementary material, table S2). Genome-wise reciprocal best hits (RBHs) of protein sequences were identified using MMseqs2 [30] (v. 13.45111, with module easy-rbh, applying a threshold of *E*-value ≤ 1 × 10^−10^). RBHs were further compared by global alignment using Parasail-python [31] (v. 1.2.4, with the Needleman–Wunsch algorithm). Sequence pairs with greater than or equal to 30% identical amino acids were clustered into protein families using the Markov clustering algorithm (MCL) [32] (v. 12-135, with parameter --abc -I 2.0).

Data analysis, statistical inference and visualization were performed with R (v. 4.0) and MATLAB^®^ (v. 2018b). Testing for a bias in ABR gene occurrence towards plasmids or chromosomes was performed by comparing the frequency of ABR gene occurrence on both replicons with the frequency of the remaining genes on both replicons (using Fisher's exact test with fisher.test function in R).

For the phylogenetic reconstruction, non-redundant protein sequences of selected ABR genes were aligned using MAFFT [33] (v. 7.475). Owing to the large number of identical protein sequences among the ABR gene homologues, the sequences were filtered to include non-redundant amino acid sequences at the resolution of replicon and genus. Phylogenetic trees were reconstructed using IQ-TREE [34] (v. 1.6.12) with restricted automatic model selection to the Le & Gascuel (LG) model [35] and the LG4X model [36] as additional alternative (with parameter -mset LG -madd LG4X). The LG model was chosen owing to its suitability for the inference of phylogenetic trees using protein sequences from bacterial organisms by means of maximum likelihood methods. The resulting trees were rooted using the midpoint criterion and visualized using iTOL [37] v. 6 (https://itol.embl.de/).

## 3. Results

### (a) Plasmid ABR gene content is associated with gene prevalence on chromosomes

The survey for genes that putatively confer ABR revealed 2810 (38%) plasmids that encode an ABR gene homologue (termed here ABR plasmids). The ABR plasmids typically encode multiple ABR genes, with the median number of ABR genes per plasmid ranging between four in *Escherichia* and six in *Salmonella*. The proportion of ABR plasmids was similar among the three genera: 34% (1131) in *Escherichia*, 42% (1256) in *Klebsiella* and 39% (423) in *Salmonella*. The genomes of all isolates in the three genera had at least one homologue to an ABR gene encoded in the chromosome.

**Figure 1.**
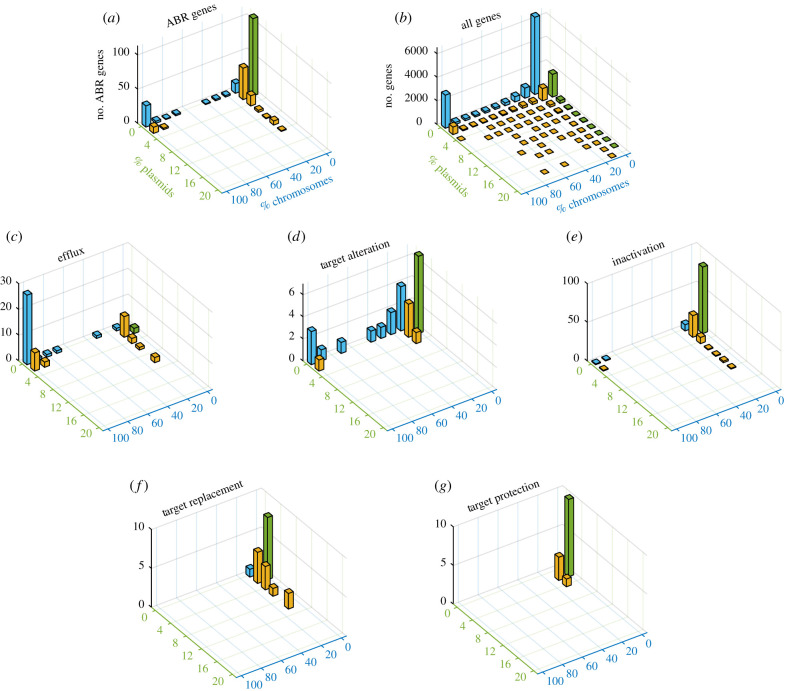
A comparison of gene prevalence in plasmids and chromosomes in *Escherichia* isolates. Plots in the figure present three-dimensional histograms of gene occurrence in plasmids (green axis) and chromosomes (blue axis). The height of bars in the histogram corresponds to the number of genes that are encoded in the same frequency group on chromosomes and plasmids. The bar coordinates correspond to the proportion of replicons (plasmids or chromosomes) where the genes occur. Note that no gene occurs in more than 20% of the plasmids; genes occurring in 100% of the chromosomes correspond to core gene families. Genes occurring only on chromosomes are shown with blue bars. Genes occurring only on plasmids are shown with green bars. Yellow bars correspond to genes that occur in both plasmids and chromosomes. (*a*) ABR genes in the examined isolates (data supplied in electronic supplementary material, table S3). (*b*) Gene occurrence of all *Escherichia* gene families. (*c*–*g*) ABR genes according to their classification into ABR mechanism (as in CARD [28]).

The ABR gene homologues in *Escherichia* plasmids and chromosomes fall within three classes: (1) ABR genes exclusively found on plasmids, (2) ABR genes found exclusively on chromosomes, and (3) ABR genes found on both plasmids and chromosomes (figure 1*a*). The pattern of gene occurrence on plasmids and chromosomes further reveals two main groups: ABR genes encoded on plasmids and shared with a small proportion of the chromosomes (less than or equal to 10%)—these genes are considered to be part of the accessory genome—and ABR genes that are encoded on plasmids and are shared with greater than or equal to 90% of the chromosomes—these genes are considered to be part of the core genome (see yellow bars in figure 1*a*). A similar pattern of ABR gene occurrence on plasmids and chromosomes is observed in the genomes of *Klebsiella* and *Salmonella* isolates (electronic supplementary material, figures S1 and S2). To compare the pattern of ABR gene occurrence with the total *Escherichia* genes, we repeated the analysis using a dataset of protein families in *Escherichia* isolates. The results show that the ABR gene occurrence in plasmids and chromosomes bears similarity to the distribution of the total gene families in *Escherichia* genomes (figure 1*b*): in both datasets homologues of core chromosomal genes found in greater than or equal to 90% of the chromosomes are less abundant in plasmids in comparison with homologues of accessory chromosomal genes found in fewer than or equal to 10% of the chromosomes. The similar pattern of shared genes among plasmids and chromosomes observed for the ABR genes and the total genome indicates that the association between ABR gene prevalence in plasmids and chromosomes is general within *Escherichia* genomes rather than a unique property of ABR genes.

Previous studies suggested that the occurrence of ABR genes in plasmids may be related to the underlying mechanism of ABR [7,24]. Indeed, further examination of the ABR gene occurrence on plasmids and chromosomes reveals differences across the ABR mechanism classes. ABR genes that are involved in the expression of efflux pumps are typically core chromosomal genes that are rarely found on plasmids (figure 1*c*). By contrast, ABR genes classified as antibiotic target alteration include both chromosome- and plasmid-exclusive genes, with rare gene sharing between plasmids and chromosomes (figure 1*d*). ABR genes classified as antibiotic inactivation comprise mostly plasmid-specific genes, with some genes occurring in chromosomes as accessory genes and rarely as core genes (figure 1*e*). Genes classified as target protection and target replacement are encoded on a small number of plasmids and chromosomes (figure 1*f*,*g*).

To further examine the mode of ABR gene evolution, that is, if it is mainly by vertical inheritance or lateral gene transfer, we explored biases in the prevalence of ABR gene homologues towards plasmids and chromosomes (termed hereafter ‘replicon type’) for each of the ABR genes in our dataset. The results of the statistical analysis confirm that most of the ABR genes coding for efflux pumps are significantly more frequent on chromosomes (table 1; electronic supplementary material, table S3). Nonetheless, some exceptional efflux-coding genes are enriched on plasmids, including *tetA/B*, *floR* and *qacE*Δ*1*, whose homologues are mainly encoded in plasmids in all three genera (electronic supplementary material, table S3). The distribution of ABR genes that encode antibiotic target alteration proteins is balanced between chromosomes and plasmids in *Escherichia*, while in *Klebsiella* and *Salmonella* it is biased towards plasmids. Examples of chromosomal antibiotic target alteration genes include the gene encoding the translation elongation factor EF-Tu, which is found as an exclusive core gene in all genera, and *bacA*, an undecaprenyl pyrophosphate phosphatase [39], which is a core chromosomal gene in *Escherichia* and *Salmonella.* Examples of genes encoding antibiotic target alteration proteins that are overrepresented on plasmids include *ermB* and *mcr-1* (electronic supplementary material, table S3).

**Table 1.** Comparison of ABR frequency in plasmids and chromosomes. The number of ABR genes having a bias towards plasmids or chromosomes according to the resistance mechanism classification (using Fisher's exact test in contingency analysis, *α* = 0.05 and correction for multiple comparisons with false discovery rate (FDR) [38]). No bias to the chromosome was found in ABR genes for antibiotic target protection and replacement (marked with ‘—’).

genus	antibiotic efflux		antibiotic inactivation		antibiotic target alteration		antibiotic target protection		antibiotic target replacement	
	chromosome	plasmid	chromosome	plasmid	chromosome	plasmid	chromosome	plasmid	chromosome	plasmid
*Escherichia*	40 (74%)	14 (26%)	3 (2%)	126 (98%)	10 (50%)	10 (50%)	—	13 (100%)	—	17 (100%)
*Klebsiella*	14 (50%)	14 (50%)	3 (3%)	100 (97%)	8 (40%)	12 (60%)	—	11 (100%)	—	13 (100%)
*Salmonella*	20 (61%)	13 (39%)	4 (5%)	74 (95%)	5 (38%)	8 (62%)	—	9 (100%)	—	13 (100%)

ABR genes in the inactivation class are typically encoded on plasmids, with rare genes showing a bias towards the chromosome (table 1). Those exceptional genes include *ampH*, which is a core chromosomal gene in all three genera. This gene encodes a penicillin binding protein that has a function in the determination of cell shape in *Escherichia coli* and can confer resistance to β-lactam antibiotics [40]. Another example is *ampC1*, which codes for a β-lactamase and is nearly universally encoded in *Escherichia* and *Salmonella* but absent in *Klebsiella* genomes. Additional examples are *aac (6’)-Iy* and *aac (6’)-Iaa*, both encoding aminoglycoside acetyltransferases, which are exclusively found in *Salmonella* chromosomes. The genes *fosA5* and *fosA6*, mediating resistance against fosfomycin, have been reported in *Escherichia* [41], yet in our extensive dataset they are observed exclusively as accessory chromosomal genes in *Klebsiella* (electronic supplementary material, table S3).

Purifying selection on plasmid gene content due to genetic conflicts is expected to differ among taxa having different compositions of their core genome. Nonetheless, the bias of ABR genes towards specific replicon type (i.e. plasmids or chromosomes) is generally similar among the three tested genera in our dataset, likely since they are closely related and their core ABR genome largely overlaps. Nonetheless, 23 ABR genes stand out as exceptions (table 2). Those are mostly plasmid genes with variable presence on the chromosomes, i.e. they can be considered accessory genes. One extreme case is the antibiotic efflux gene *oqxA*, which is a core chromosomal gene in *Klebsiella* and enriched on plasmids in the other two genera. Among the exceptional genes encoding antibiotic inactivation proteins, the gene *bla*_CARB-1_ is found as an accessory chromosomal gene in *Salmonella*, with no bias towards specific replicon type, but only on plasmids in *Escherichia* and *Klebsiella* (electronic supplementary material, table S2).

**Table 2.** ABR genes whose relative frequency in plasmids (Pls) and chromosomes (Chr) differs among the three tested genera. The table shows ABR gene occurrence on plasmids and chromosomes in the three genera. The ABR gene frequency on both replicons was compared using Fisher's exact test (the *p*-value is reported in addition to adjusted *p*-value for multiple comparisons using false discovery rate (FDR)). Chromosome- and plasmid-exclusive genes and genes absent from any of the genera were excluded from this analysis (a total of 98 ABR genes were tested). Genes discussed in the Results §§3(*c*)–3(*e*) are highlighted in italics.

		resistance mechanism	*Escherichia*		*Salmonella*		*Klebsiella*			
ARO	gene		Chr	Pls	Chr	Pls	Chr	Pls	*p*	*p*_adj_
*3003922*	*oqxA*	antibiotic efflux	2	45	2	36	728	1	1.02 × 10^−105^	1.00 × 10^−103^
*3003923*	*oqxB*	antibiotic efflux	2	31	2	19	162	0	5.51 × 10^−45^	2.70 × 10^−43^
*3000165*	*tetA*	antibiotic efflux	62	283	20	165	13	232	1.02 × 10^−5^	7.71 × 10^−5^
*3000166*	*tetB*	antibiotic efflux	69	100	39	58	3	6	9.47 × 10^−1^	1.00 × 10^−1^
*3005010*	*qacE*Δ*1*	antibiotic efflux	55	291	68	137	116	371	1.73 × 10^−5^	1.21 × 10^−4^
*3000873*	*bla_*TEM-1*_*	antibiotic inactivation	65	333	53	133	9	404	6.65 × 10^−22^	2.17 × 10^−20^
*3004290*	*ampC*	antibiotic inactivation	1119	1	2	0	0	0	—	—
*3003689*	*mcr-1*	antibiotic target alteration	13	95	0	26	0	8	1.10 × 10^−1^	2.83 × 10^−1^
*3002790*	*qnrS1*	antibiotic target protection	8	105	1	41	2	132	6.61 × 10^−2^	2.06 × 10^−1^
*3000412*	*sul2*	antibiotic target replacement	90	287	55	187	21	272	2.00 × 10^−9^	2.79 × 10^−8^
*3002705*	*floR*	antibiotic efflux	33	143	33	121	2	63	9.17 × 10^−4^	4.99 × 10^−3^
*3000168*	*tetD*	antibiotic efflux	6	1	1	4	2	50	3.69 × 10^−6^	3.28 × 10^−5^
3002660	*aph(6)-Id*	antibiotic inactivation	82	270	60	173	17	249	1.70 × 10^−10^	4.16 × 10^−9^
3002639	*aph(3″)-Ib*	antibiotic inactivation	82	263	59	167	18	245	4.26 × 10^−10^	8.34 × 10^−9^
3001059	*bla*_SHV-1_	antibiotic inactivation	0	2	0	1	144	7	2.01 × 10^−4^	1.23 × 10^−3^
3002601	*aadA*	antibiotic inactivation	43	130	10	76	3	133	8.62 × 10^−9^	1.06 × 10^−7^
3001877	*bla*_CTX-M-14_	antibiotic inactivation	23	43	2	13	7	51	7.64 × 10^−3^	3.57 × 10^−2^
3002013	*bla*_CMY-2_	antibiotic inactivation	22	42	6	70	0	7	1.81 × 10^−4^	1.18 × 10^−3^
3002539	*aac(3)-IV*	antibiotic inactivation	5	37	4	45	12	21	4.48 × 10^−3^	2.20 × 10^−2^
3002603	*aadA3*	antibiotic inactivation	5	29	27	29	97	44	1.70 × 10^−8^	1.85 × 10^−7^
3002683	*catI*	antibiotic inactivation	35	27	26	35	9	81	5.23 × 10^−1^	8.54 × 10^−9^
3001926	*bla*_CTX-M-65_	antibiotic inactivation	4	13	1	20	0	76	5.21 × 10^−4^	3.00 × 10^−3^
3004656	*catII*	antibiotic inactivation	0	7	4	19	1	88	8.84 × 10^−3^	3.94 × 10^−2^
3002240	*bla*_CARB-1_	antibiotic inactivation	0	4	25	0	0	2	1.36 × 10^−6^	1.33 × 10^−5^
3001397	*bla*_OXA-2_	antibiotic inactivation	3	0	0	1	0	0	2.75 × 10^−3^	1.42 × 10^−2^
3000410	*sul1*	antibiotic target replacement	53	286	66	140	76	371	8.05 × 10^−6^	6.58 × 10^−5^
3002862	*dfrA7*	antibiotic target replacement	13	2	21	23	0	1	9.83 × 10^−3^	4.19 × 10^−2^

### (b) ABR gene transfer between plasmids and chromosomes

Our results reveal 133 (33%) ABR genes that are found on both replicons in at least one genus, thus indicating that they have been laterally transferred. Of those, 75 ABR genes were observed on both plasmid and chromosome within the same isolate genome in at least one strain. The majority of genes that we identified at least once as duplicated are enriched on plasmids (65 out of 75 genes); of those, 45 genes encode proteins that function in antibiotic inactivation (electronic supplementary material, table S4). Only five of the 75 duplicated genes are enriched for a chromosome location; these correspond to four genes encoding efflux pumps and one encoding translation elongation factor (target alteration). The presence of duplicated ABR genes on both replicons in the same isolate is relatively rare. We note here that deleterious effects in the context of gene gain following lateral gene transfer [23] are expected not only for the recipient, but rather also for the donor, following gene transfer from chromosomes to plasmids. Taken together, our results suggest that genes that are duplicated on the plasmid and chromosome in the same isolate often correspond to genes introduced into the genus as plasmid genes.

To further study the evolutionary history of genes shared between plasmids and chromosomes we reconstructed the phylogenetic trees of the ABR genes and examined their topology. In what follows, we present the results of 39 selected ABR genes exemplifying main trends in the data. The topology of ABR genes across replicon types and genera in the inferred phylogenetic trees confirms the occurrence of lateral gene transfer among genera and also replicon types in the history of all examined genes (electronic supplementary material, table S5). In the following, we present several prime examples.

### (c) Lateral transfer of genes encoding efflux pumps

**Figure 2.**
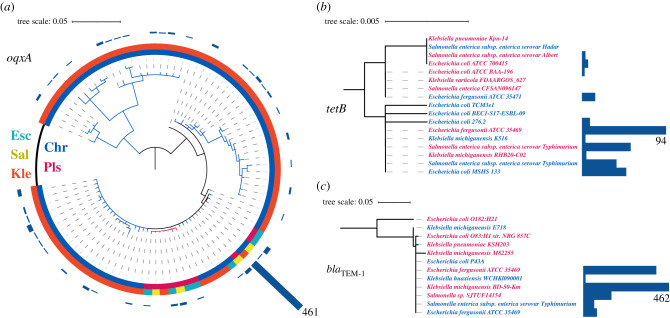
Lateral gene transfer in the evolution of ABR genes. Bars next to the operational taxonomic units show the frequency of redundant amino acid sequences (with highest frequency noted). (*a*) The outer ring shows the genus of ABR gene origin; the inner ring shows whether the gene is encoded on a plasmid (Pls) or chromosome (Chr). (*b*,*c*) Isolate label is coloured according to replicon type (plasmid genes in pink and chromosomal genes in blue). Most phylogenetic trees we reconstructed here for the ABR genes included polytomies, hence donor and recipients in the gene transfer events cannot be reliably inferred. Nonetheless, considering the presence of the examined ABR genes on both plasmids and chromosomes supports the hypothesis that they have been transferred at least once in their evolutionary history.

**Figure 3.**
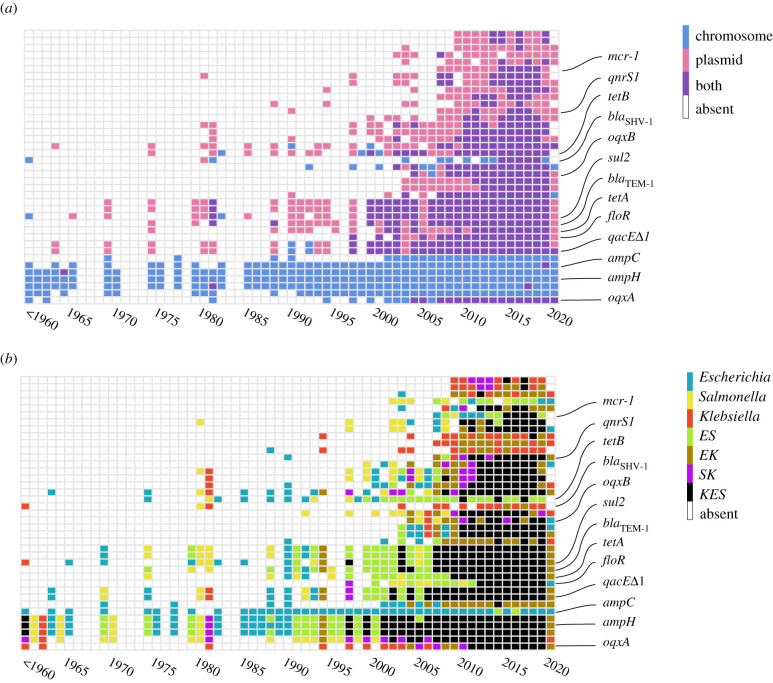
Temporal pattern of ABR gene documentation in replicons and genera. Gene presence according to the isolation date of the strains is summarized. The information on the strains isolated between 1900 and 1960 is condensed in ‘<1960’. The figure shows data for 39 genes whose phylogenetic trees were closely examined (ordered as in electronic supplementary material, table S4). The names of 13 genes highlighted in the manuscript are marked. Different colours correspond to gene locations. (*a*) Temporal ABR gene pattern according to replicon type. (*b*) Temporal ABR gene pattern according to genus. ABR genes found in multiple genera are annotated by capitalized abbreviations of the three genera, i.e. *E* for *Escherichia*, *K* for *Klebsiella, S* for *Salmonella.*

The genes *oqxA* and *oqxB* belong to the resistance–nodulation–cell division (RND) family of bacterial efflux pumps [42] and were first isolated from bacteria in swine manure from a farm where olaquinodox was used as a feed additive [43]. The proteins OqxA and OqxB, along with TolC, form an efflux pump that provides resistance against olaquinodox, chloramphenicol, quinolones, trimethoprim, tigecycline, nitrofurantoin, and also several detergents and disinfectants by reducing their concentrations in the cell [42,44]. Both *oqxA* and *oqxB* are almost strictly chromosomal genes in *Klebsiella*, with *oqxA* occurring in 86% and *oqxB* occurring in 19% of *Klebsiella* isolates. We identified a single *Klebsiella* isolate where *oqxA* is present on both chromosome and plasmid (electronic supplementary material, table S4). The two genes have homologues in *Escherichia* and *Salmonella* plasmids. The phylogenetic trees of both genes indicate lateral transfer between *Klebsiella* chromosomes and plasmids of the other two genera (figure 2*a*; electronic supplementary material, figure S3*a*). We note that the *oqxA* protein sequences are nearly identical, and hence the phylogenetic reconstruction does not allow a reliable inference of donors and recipients in the transfer event. To further study the history of the ABR genes, we collected information on the isolation date of strains included in our analysis. The gene *oqxA* was documented early in the 1950s as a chromosomal gene in *Klebsiella* (figure 3). The first documentation of *oqxA* on a plasmid in our dataset is from 2004 in *Escherichia*; in recent years, this gene has been found on both plasmids and chromosomes in all three genera. The gene *oqxB* was first documented in 2004 in *Klebsiella* chromosomes and *Escherichia* plasmids, while in recent years, it has been described for both replicon types in all three genera (figure 3). The example of *oqxA* suggests that barriers to gene transfer of core chromosomal genes may differ between genera. The first report on plasmid-encoded *oqxAB* in *E. coli* in the literature dates back to the late 1990s [45]; today, after more than 20 years, that gene is still very rare in *Escherichia* and *Salmonella*. It is tenable to speculate that barriers to plasmid-mediated *oqxA* acquisition are present also in *Escherichia* and *Salmonella*.

The Tet efflux pumps were the first efflux systems discovered in bacteria [46,47]. These major facilitator (MF) family exporters are membrane–associated proteins that pump out tetracycline from the cell in an energy-dependent manner [48]. Unlike the OqxAB–TolC system, the Tet efflux pumps provide resistance only against tetracyclines. Since the original report of *tetA*, many more *tet* genes have been discovered, the vast majority in Gram-negative bacteria [49]. For our analysis, we focused on the *tetA* and *tetB* genes, since both genes are enriched on plasmids and rarely found on chromosomes (fewer than 6% in all genera). The phylogenetic reconstruction shows that the protein sequence of *tetA* is more diverged in comparison with that of *tetB*, and that for both genes, multiple transfer events occurred between chromosomes and plasmids, and also across bacterial genera (figure 2*b*; electronic supplementary material, figure S3*d*). Furthermore, we identified 13 isolates (from all genera) where *tetA* is encoded on both replicon types and four *Escherichia* isolates that encode *tetB* on both replicon types. The gene *tetA* in our data was documented firstly on a plasmid in *Salmonella* in 1974, while *tetB* was identified on a plasmid in *Escherichia* at the same time (figure 3). In recent years, both genes have been found on both replicon types and in all three genera, yet *tetB* is seldom documented in *Klebsiella*.

Another efflux gene that is mainly found on plasmids is *qacE*Δ*1*, a truncated variant of the *qacE* gene [50]. The *qac* genes are antiseptic efflux systems primarily exporting quaternary ammonium compounds. Their substrate range, however, is quite large and includes intercalating dyes, biguanidines, diamidines and xanthenes. The gene *qacE*Δ*1* is frequently found in ABR gene cassettes isolated from bacteria exposed to these compounds compared with those that are not [51]. In our dataset, we found that *qacE*Δ*1* is very often encoded on both plasmids and chromosomes in the same isolate (71 *Klebsiella*, 11 *Escherichia* and 7 *Salmonella* isolates). The protein sequence is highly conserved between replicons and across all three genera. In our dataset, *qacE*Δ*1* was documented first on a plasmid in *Escherichia* in 1969, and then a *Klebsiella* plasmid in 1981; the first chromosomal *qacE*Δ*1* was identified in *Escherichia* in 1990 (figure 3).

### (d) Lateral transfer of genes encoding antibiotic inactivation proteins

A prime example of antibiotic inactivation enzymes are the β-lactamases. These enzymes confer resistance to β-lactam antibiotics by cleaving their central β-lactam ring [52]. The gene *bla*_TEM-1_ was the first TEM β-lactamase gene identified;TEM β-lactamases are among the best-studied ABR enzymes [53,54]. Although the original *bla*_TEM-1_ gene confers resistance only to penicillins and early cephalosporins, its later variants also provide resistance against second-, third- and fourth-generation cephalosporins, monobactams and β-lactamase inhibitors [54]. The *bla*_TEM-1_ gene is mainly found on *Klebsiella* and *Escherichia* plasmids. The phylogenetic reconstruction indicates multiple transfer events and high protein sequence conservation of *bla*_TEM-1_ between replicons and across genera (figure 2*c*). It was first documented on plasmids in both *Escherichia* and *Salmonella* in 1974, and first reported on chromosomes in *Salmonella* in 1981 (figure 3).

The AmpC β-lactamase of *Escherichia coli* was the first reported bacterial enzyme capable of degrading penicillin [55]. Although active against penicillins, this inducible enzyme has higher activity against cephalosporins and is an important determinant of resistance against cephalosporins in clinical settings [56]. The *ampC* gene in *Escherichia* is nearly exclusively a chromosomal gene. Additionally, homologues of *ampC* were found on a plasmid in one *Escherichia* isolate and on the chromosome of two *Salmonella* isolates. The phylogenetic reconstruction indicates that the *Salmonella* homologues were acquired in two independent transfer events (electronic supplementary material, figure S3*h*).

### (e) Lateral transfer of ABR genes involved in target alteration, replacement and protection

The *mcr-1* gene was the first plasmid-mediated colistin resistance determinant reported, signalling the breach of the last resort polymyxin antibiotics [57]. MCR-1 is a phosphoethanolamine transferase that alters lipid A in the outer membrane of Gram-negative bacteria, resulting in reduced affinity to colistin [58]. Homologues of MCR-1 are mainly found on plasmids in all three genera, with some rare homologues on chromosomes in *Escherichia* (1%). Three *Escherichia coli* isolates were identified with *mcr-1* on both replicons. The nearly identical protein sequence of the homologues suggests that *mcr-1* is laterally transferred among the genera by plasmids (electronic supplementary material, figure S3*e*). The gene was first documented on a plasmid in *Escherichia* in 2007 and on the chromosome in 2014; in recent years it has been found in all three genera (figure 3).

The gene *sul2* confers resistance against the sulfonamide antibiotics [59,60]. Sulfonamides were among the first synthetic compounds for which specific antibacterial activities were discovered, and they were also among the first antibiotics to be used for medical treatment. They block folic acid synthesis in bacteria by competitively inhibiting dihydropteroate synthase (DHPS) [61]. Sul2 is an alternative drug-resistant variant of the DHPS enzyme that replaces the original drug-sensitive variant to provide resistance [62]. The gene is mainly found on plasmids and may occur also on chromosomes (6% of isolates). A total of 34 isolates encode a *sul2* on both replicons, and the phylogenetic tree of *sul2* indicates multiple transfer events between replicons and across genera (electronic supplementary material, figure S3*f*). Although typically a plasmid gene, *sul2* was first documented on the chromosome in *Klebsiella* in the 1950s and only later on plasmids in *Escherichia* and *Salmonella*; in recent years, it has been reported in all three genera (figure 3).

The gene *qnrS1* belongs to the Qnr family of proteins, which confer resistance to quinolones and flouroquinolones in Gram-negative bacteria by binding and protecting the target of these antibiotics, type II topoisomerase [63]. The binding of the target seems to destabilize the complex of topoisomerase with the drug, resulting in regeneration of the active enzyme [64]. Homologues of *qnrS1* are mainly distributed on plasmids. We identified two isolates that carry this gene on both replicons, including *E. coli* strain LHM10-1 (isolated in 2017 in China from swine faeces) and *Klebsiella pneumoniae* strain C2421 (isolated in 2017 in China from human urine sample). The QnrS1 protein sequences are nearly identical in all plasmids and the phylogenetic tree topology indicates lateral gene transfer between replicons and in all three genera (electronic supplementary material, figure S3*g*). The gene was first documented on plasmids in *Escherichia* and *Klebsiella* in 2007 and in recent years it has been found on both replicons in all three genera (figure 3).

### (f) Temporal pattern of gene presence in replicons supports the phylogenetic inference

To further study the temporal pattern of ABR gene occurrence on specific replicon type—plasmids or chromosomes—we examined the collection date of isolate genomes encoding ABR genes. The available data reveal two general trends: core ABR genes were typically documented on chromosomes, with only rare findings on plasmids throughout the time range covered by the data. Notably, of the 75 core ABR genes that are enriched on chromosomes, 74 (99%) were documented first on chromosomes, with *oqxB* as the sole exception. Additionally, of the 226 genes found to be enriched on plasmids, 208 (92%) were first identified on plasmids. The temporal pattern of plasmid genes shows a trend of early documentation on plasmids and later presence on both replicons, which is in agreement with the phylogenetic trees reconstructed in this study (figure 2). The temporal pattern of ABR gene occurrence as observed in isolate genomes provides further support for the role of historical contingency in the bias of ABR gene occurrence on plasmids or chromosomes.

## 4. Discussion

In this study, we performed a comprehensive ABR gene analysis across 2635 complete genome sequences in the related bacterial genera *Escherichia*, *Salmonella* and *Klebsiella*, and identified an inverse association between ABR gene prevalence in plasmids and chromosomes. This inverse association strongly suggests that the presence of a homologous gene in the chromosome poses a barrier to plasmid-mediated ABR gene acquisition. Several non-exclusive mechanisms may account for the barrier to lateral gene transfer, as explained in detail below.

The bias of ABR genes towards specific replicon type—chromosomes or plasmids—is clearly associated with the ABR underlying mechanism [7]. ABR genes associated with efflux pumps are primarily encoded on chromosomes, those involved in target alteration can be encoded on both replicon types, and those for the remaining three mechanisms are almost exclusively encoded on plasmids. ABR genes encoding efflux pumps are frequently part of the core genome (e.g. in the genera we studied here), where they code for proteins having multiple functions in the cell outside the context of ABR. Examples are proteins involved in export of heavy metals [65], organic pollutants [66] and endogenous metabolites [67], and cell signalling molecules [68]. Furthermore, several efflux pumps are important for biofilm formation [69] and bacteria–plant interactions [70]. Core genes are expected to be highly integrated in the cellular transcriptional regulation network and their products are likely well integrated in protein–protein interaction networks. Proteins encoded by the core genome are therefore expected to have a high level of pleiotropic effects on bacterial physiology. Barriers for lateral transfer of core genes are expected to be high, e.g. owing to genetic conflicts [7] or the cost of their interference with well-coordinated cellular processes [23]. The same logic applies to core genes in the other classes of resistance mechanisms, including target alteration and antibiotic inactivation. Core ABR genes that code for antibiotic target alteration in our dataset include *gyrA*, *parC* and *parE.* These genes may provide resistance to antibiotics that target, e.g., type II topoisomerases, DNA gyrase and topoisomerase IV, which control topological transitions of DNA [71]; all of these are central components in the bacterial information processing mechanisms that are targets for drug therapy (e.g. with fluoroquinolones [4]). The frequency of ABR gene transfer in microbial evolution (i.e. transferability) is therefore expected to depend on the complexity of their function within the cell [18,19] rather than the exact mechanism of resistance, similarly to other genes that do not code for ABR mechanisms.

So far, we have discussed barriers to lateral transfer of ABR genes depending on the effect of gene acquisition on the bacterial organism. Notwithstanding, the acquisition of ABR genes may have consequences also for the evolution of stably inherited plasmids. Plasmids encoding ABR genes may also evolve a stable inheritance in a newly colonized host population in the absence of selective conditions for ABR [72]. By contrast, the presence of selective conditions for ABR can maintain in the population plasmids whose inheritance is unstable; such plasmids are at risk of extinction under non-selective conditions [73]. Indeed, plasmids equipped with mobility mechanisms have the capacity to persist via transfer to alternative host populations [74]. Nonetheless, the stability of plasmid vertical inheritance is paramount for plasmid survival and long-term evolution (reviewed in [75]). The potential deleterious effect of ABR gene acquisition in plasmid genomes on the plasmid fitness [76] may explain the rarity of ABR genes on plasmids (figure 1), specifically in non-mobile plasmids [73].

Plasmid-encoded ABR genes in our dataset are mostly classified as antibiotic target inactivation (figure 1). ABR genes coding for antibiotic inactivation frequently vary in their relative frequency on plasmids and chromosomes across the three compared genera. Many of those genes correspond to accessory chromosomal genes that are also prevalent on plasmids, indicating that they may be transferred between both replicon types (table 2). Indeed, accessory genes may prove essential depending on the isolate genetic background and environmental conditions [77]; nonetheless, their variable presence in a genus suggests that they are often dispensable. Genes whose evolution is characterized by rapid gain and loss dynamics are expected to encode proteins whose function is weakly dependent on specific genetic backgrounds, and are therefore more likely to evolve by lateral gene transfer [78]. The ABR gene content of plasmids reflects the propensity of specific ABR genes to be successfully transferred among donors and recipients within the plasmid host range.

## Data accessibility

 The data are provided in electronic supplementary material [79].
